# Identification of drought tolerant Chickpea genotypes through multi trait stability index

**DOI:** 10.1016/j.sjbs.2021.07.056

**Published:** 2021-07-24

**Authors:** Tamoor Hussain, Zahid Akram, Ghulam Shabbir, Abdul Manaf, Mukhtar Ahmed

**Affiliations:** a Department of Plant Breeding and Genetics, PMAS Arid Agriculture University, Rawalpindi 46300, Pakistan; b Department of Agronomy, PMAS-Arid Agriculture University, Rawalpindi 46300, Pakistan; c Department of Agricultural Research for Northern Sweden, Swedish University of Agricultural Sciences, 90183 Umeå, Sweden

**Keywords:** Multi-environment trials, Multi-trait stability index, Best Non-Impartial Linear Forecast, Drought, Chickpea

## Abstract

Drought is a major and constantly increasing abiotic stress factor, thus limiting chickpea production. Like other crops, Kabuli Chickpea genotypes are screened for drought stress through Multi-environment trials (METs). Although, METs analysis is generally executed taking into account only one trait, which provides less significant reliability for the recommendation of genotypes as compared to multi trait-based analysis. Multi trait-based analysis could be used to recommend genotypes across diverse environments. Hence, current research was conducted for selection of superior genotypes through multi-trait stability index (MTSI) by using mixed and fixed effect models under six diverse environments. The genotypic stability was computed for all traits individually using the weighted average of absolute scores from the singular value decomposition of the matrix of best linear unbiased predictions for the genotype vs environment interaction (GEI) effects produced by a linear mixed-effect model index. A superiority index, WAASBY was measured to reflect the MPS (Mean performance and stability). The selection differential for the WAASBY index was 11.2%, 18.49% and 23.30% for grain yield (GY), primary branches per plant (PBP) and Stomatal Conductance (STOMA) respectively. Positive selection differential (0.80% ≤ selection differential ≤ 13.00%) were examined for traits averaged desired to be increased and negative (-0.57% ≤ selection differential ≤ -0.23%) for those traits desired to be reduced. The MTSI may be valuable to the plant breeders for the selection of genotypes based on many characters as being strong and simple selection process. Analysis of MTSI for multiple environments revealed that, the genotypes G20, G86, G31, G28, G116, G12, G105, G45, G50, G10, G30, G117, G81, G48, G85, G17, G32, G4, and G37 were the most stable and high yielding out of 120 chickpea genotypes, probably due to high MPS of selected traits under various environments. It is concluded that identified traits can be utilized as genitors in hybridization programs for the development of drought tolerant Kabuli Chickpea breeding material.

## 1 Introduction

The mean global temperatures have risen to record 1.2 ˚C higher than previous century (Voosen, 2021). Likewise, it has been predicted that in 2100 it will further increase and could go up to 3 ˚C (Schneider et al., 2007). Many scientists projected that drought, higher magnitude of concentration of CO_2_ and global temperatures will go higher with the passage of time in sub-tropical and semi-arid ecological zones (Araus et al., 2002, Wang et al., 2017). Consequently, due to these variations the rate of crop evapotranspiration will increase which could create more worse scenario for crop production due to lesser water availability to fulfill crop water needs (Abbas et al., 2017, Ahmad et al., 2017, Ahmad et al., 2019, Fatima et al., 2020, Fatima et al., 2021). This will lead to high risk of lesser crop production (Ahmed et al., 2020, Ahmed, 2020, Zampieri et al., 2020) and consequently declined water productivity (Ali et al., 2020, Amiri et al., 2021). Determinantal impact of climate change and drought on food and water security have been reported by Ding et al. (2021). Their results suggested that these problems could be solved by using different management options through simulation modeling.

Chickpea (*Cicer arientinum* L.) is the 3rd largely grown pulse crop across the globe with harvested area of 13.72 million hectare and production of 14.25 million tons in 2019. Asia is the largest chickpea producer with production of 90.6% followed by Americas (5.2%), Africa (3.5%), Europe (0.6%) and Oceania (0.1%) (FAOSTAT, 2019). It is the 2nd most important legume crop after common bean (Gaur et al., 2008). The major producer of chickpea includes India, Australia, Canada, Ethiopia, Iran, Mexico, Myanmar, Pakistan, Turkey, and the USA (Dixit et al., 2019). Chickpea have two main types i.e. desi and kabuli and it has been suggested that desi × kabuli introgressions could be used for improving the adaptability and yield stability of Kabulis (Purushothaman et al., 2014). Chickpea is an important source of protein for mankind with protein content of 16 to 28% (Liu et al., 2008, Chibbar et al., 2010). Whole chickpea have leucine and lysine which are the most abundant essential amino acids. It also has sulfur containing amino acids i.e. cysteine and methionine in limited amount (Wang et al., 2010). Chickpea is a drought tolerant legume crop while worldwide production entails due to its multiple use as food, feed, fuel and fertilizer (Gaur et al., 2010, Devasirvatham and Tan, 2018). Although, chickpea can tolerate drought stress but significant negative effects on the productivity of chickpea have been reported due to drought (Jha et al., 2014, Rani et al., 2020, Shah et al., 2020). Thus it is essential to have resilient crop cultivars so that crop can have potential to endure water stress period (Massawe et al., 2015). Drought impacts on crop have been further worsen due to climate change (Thomas, 2008, Korres et al., 2016, Tripathi et al., 2016). Since climate change resulted to spatio-temporal variability in the rainfall intensity and distribution thus it is the main cause of water stress across the globe (Vergni and Todisco, 2011, IPCC, 2014, Nicholson et al., 2018, Talchabhadel et al., 2018, Caloiero et al., 2019, Jamro et al., 2019, Ahmed, 2020, Mengistu et al., 2020, Yang et al., 2021). Furthermore, to fulfill the protein demand of increasing human population it is essential to increase the productivity of chickpea on long term basis (Chaturvedi et al., 2018).

Major abiotic challenges faced by chickpea production includes drought, low and high temperature at different growth stages (Jha et al., 2014, Garg et al., 2015). Moreover, unpredictable climate change is the top most constraint which leads to climate extreme events with higher frequency of drought and temperatures (Low < 15 °C and High greater than 30 °C) that reduces grain yields significantly (Kadiyala et al., 2016). Hence, it is imperative to identify and develop high-stable yielding varieties of Chickpea to coup abiotic stress which has been main task of this work (Devasirvatham and Tan, 2018).

Drought is a foremost and constantly increasing abiotic stress that limits crop production across the world (Vurukonda et al., 2016). It is the most destructive abiotic stress affecting world’s food security. The negative effect of drought appears significantly in the arid and semi-arid regions (Murungweni et al., 2016, El Sayed et al.,, Kheir et al., 2021). It has been documented that crop growth at more than 50% of the arable lands will be significantly affected by the drought (Vinocur and Altman, 2005). Drought stress under changing climate and ever-increasing population is serious concern for agriculture. Severe drought has shown negative effects on crop growth, development, and yield (Barnabás et al., 2008, Makonya et al., 2020, Cohen et al., 2021, Jabbari et al., 2021, Waqas et al., 2021). Annually 40–50% reduction in yield across globe is reported due to terminal drought (Ahmad et al., 2005, Thudi et al., 2014). Improvement in drought tolerance is possible by understanding various morphological, physiological and biochemical responses to drought stress (Shah et al., 2020). Similarly, different agronomic managements and development of new plant types are recommended to meet the main challenges of chickpea adaptation to stresses (Vadez et al., 2021). A significant positive correlation with grain yield, high heritability coupled with high genetic variability and less yield losses under optimal conditions are essential for a character to be expressed as drought tolerance marker (Maqbool et al., 2017, Chandora et al., 2020). Therefore, a comprehensive multiple enhancement approach is needed for sustainable crop production under drought stress (Arif et al., 2021). Thus, there is dire need to utilize techniques those present adaptability as well as stability to select the most excellent genotypes under different environmental conditions. The different breeding techniques used to enhance the drought tolerance in chickpea would be applied through integration of morphological and physiological systems of drought tolerance from resistant genotypes (Kumar et al., 2020, Singh et al., 2021). Numerous physiological, phenological and morphological characters have been established those play an imperative role in adaptation of a particular crop in adverse environmental conditions (Najan et al., 2018, Sharifi et al., 2018).

Multi environment trials (MET) are mostly used to evaluate impact of drought stress on crops could be analyzed by using additive main effect and multiplicative interaction (AMMI) and best linear unbiased prediction (BLUP) methods (Olivoto et al., 2019a, Olivoto et al., 2019b). AMMI stability and drought tolerance indices were recommended as drought-tolerance evaluation methods particularly in resource poor countries (Arif et al., 2021). The AMMI is good graphical tool, but it lacks linear mixed‐effect model (LMM) while BLUP provides good estimates, however, new insights are needed to deal with a random genotype vs environment interaction (GEI). The Weighted Average of Absolute Scores (WAASB) is a new quantitative genotypic stability measure that could be used to address above mentioned issues. This could help agronomists and breeders to make correct decisions for selection and recommendations of specific genotypes. Furthermore, MET analysis is performed on the basis of single trait, mainly keeping in view the grain yield only (Nowosad et al., 2016, MOHAMMADI et al., 2018, Erdemci, 2018, Azam et al., 2020, Shah et al., 2020). Conversely, the more reliable genotypes can be selected when multi traits are considered in MET analysis under different environments. For this purpose, a technique for MET analysis (METAN) allows for the comprehensive selection for MPS of numerous traits into a single index could provide a unique selection process. Multi‐trait stability index (MTSI) is valuable to the plant breeders for the selection of genotypes based on several traits as it gives a strong and simple selection process (Olivoto et al., 2019a, Olivoto et al., 2019b). These tools have been used successfully for the selection of drought and salinity tolerant soyabean genotypes (Zuffo et al., 2020) and determination of quality traits in *Brassica* spp. Genotypes (Bocianowski et al., 2018). The work of Zuffo et al., 2020, Bocianowski et al., 2018, Nowosad et al., 2016 successfully selected the crop genotypes on the basis of multi-traits under diverse climatic conditions. Thus, present study was designed with the goal to identify the most stable and high yielding chickpea genotypes by applying popularly recommended multi-trait stability analysis under diverse environmental conditions.

## 2 Materials and methods

### 2.1 Multi location experiments

**Fig. 1:**
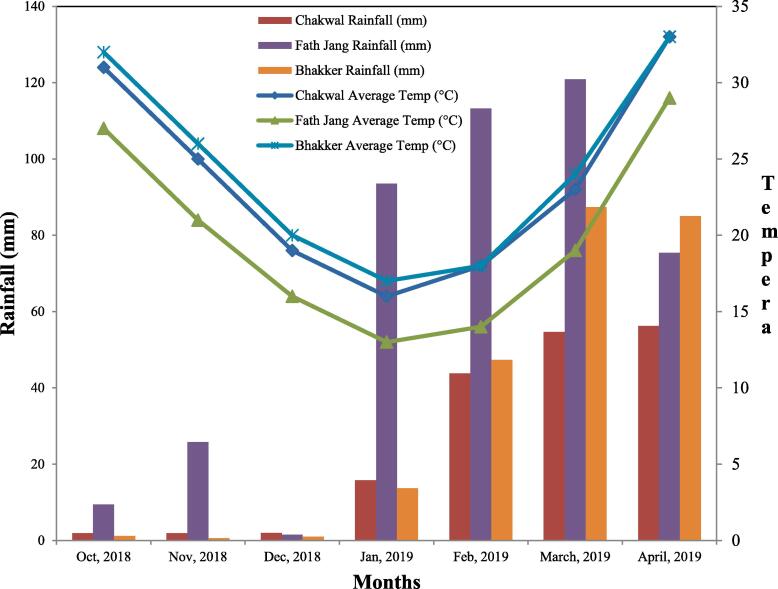
Rainfall (mm) and average Temperature (^o^C) data on monthly basis for different environments under studied.

The current research work was conducted by using 120 Kabuli chickpea genotypes including three local checks collected from different Research Institutes in Pakistan (Table 1). To assess the performance and stability simultaneously, these Kabuli chickpea genotypes were evaluated in six environments (E) (E1: Chakwal full irrigated, E2: Chakwal limited irrigation, E3: Chakwal drought stress, E4: Chakwal rainfed, E5: Bhakkar rainfed and E6: Fateh Jang rainfed conditions) to select the superior genotypes based on multiple traits during Rabi season 2018–19. The field research was performed in alpha lattice design comprised of two replications at each environment. Chakwal full irrigated (E1), limited irrigation (E2) and drought stress (E3) were developed at Barani Agricultural Research Institute, Chakwal by managing three complimentary irrigation treatments viz: T1 (Control); a well irrigated treatment, T2 (Limited Irrigation): Irrigation at the field bed preparation, flowering initiation, and pod development, T3 (Drought stress): Irrigation at the field bed preparation and at flowering initiation only respectively under rain shelter conditions. While others three environments (E4, E5 and E6) relates to rainfed conditions under different environments dependent purely on the seasonal rainfall. The rainfall (mm) and average temperature (^0^C) on monthly basis is presented in Fig. 1. For comparison of mean performance and stability analysis approved varieties CM-2008, Noor-2013 and Tamman were used as check. The plant-to-plant distance was kept 30 cm while row to row distance was maintained to 15 cm using hand drill sown in the last week of October at three sites. The recommend dose of fertilizer was used i.e. 25 kg N ha^−1^, 90 kg P ha^−1^ and 30 kg K ha^−1^ at the time of final seedbed preparation. Weeding and other management practices were adopted same under all environments except for the provision of supplementary irrigation where required as treatment. The data for DTF: Days to fifty percent flowering, DTM: Days to Maturity, PH: Plant height in cm, PBP: Number of primary branches per plant, PPP: Number of Pods per plant, SPP: Number of Seeds per pod, HSW: 100 seed weight, HI: Harvest index %, BY: Biological yield Kg ha^−1^, GY: Grain yield Kg ha^−1^, PHOTO: Photosynthesis rate, µmoles CO_2_ m^−2^ *sec*^-1^, STOMA: Stomatal conductance, Mol H_2_O m^−2^ *sec*^-1^, CHLOR: Chlorophyll contents, mg g^−1^ fresh weight, TRANS: Transpiration rate, mMol H_2_O m^−2^ *sec*^-1^, INT. CO_2_: Intercellular CO_2_ concentration, vpm and WUE: Water use efficiency were recorded using standard protocols.

**Table 1:** The Kabuli chickpea genotypes collected from various research institutes in Pakistan.

Sr. Code	Genotype No.	Origin	Sr. Code	Genotype No.	Origin	Sr. Code	Genotype. No	Origin
G1	17KCC-101	BARI	G41	13KCC-114	BARI	G81	6KCC-103	BARI
G2	17KCC-105	BARI	G42	13KCC-115	BARI	G82	6KCC-121	BARI
G3	17KCC-106	BARI	G43	13KCC-116	BARI	G83	6KCC-124	BARI
G4	17KCC-107	BARI	G44	12KCC-101	BARI	G84	6KCC-126	BARI
G5	17KCC-108	BARI	G45	12KCC-103	BARI	G85	09AG-15	AZRI
G6	17KCC-109	BARI	G46	12KCC-104	BARI	G86	09AG-37	AZRI
G7	17KCC-114	BARI	G47	12KCC-105	BARI	G87	11AG-38	AZRI
G8	17KCC-115	BARI	G48	12KCC-106	BARI	G88	11AG-41	AZRI
G9	17KCC-116	BARI	G49	12KCC-108	BARI	G89	11AG-43	AZRI
G10	17KCC-117	BARI	G50	12KCC-109	BARI	G90	11AG-48	AZRI
G11	17KCC-118	BARI	G51	12KCC-110	BARI	G91	Aus Sel-100	BARI
G12	16KCC-101	BARI	G52	12KCC-111	BARI	G92	Aus Sel-101	BARI
G13	16KCC-105	BARI	G53	12KCC-112	BARI	G93	Aus Sel-102	BARI
G14	16KCC_106	BARI	G54	12KCC-119	BARI	G94	12AG-56	AZRI
G15	16KCC-107	BARI	G55	12KCC-120	BARI	G95	12AG-60	AZRI
G16	15KCC-101	BARI	G56	11KCC-112	BARI	G96	12AG-61	AZRI
G17	15KCC-106	BARI	G57	11KCC-113	BARI	G97	12AG-129	AZRI
G18	15KCC-107	BARI	G58	11KCC-114	BARI	G98	12AG-133	AZRI
G19	15KCC-110	BARI	G59	11KCC-115	BARI	G99	12AG-230	AZRI
G20	15KCC-112	BARI	G60	11KCC-119	BARI	G100	12AG-235	AZRI
G21	15KCC-113	BARI	G61	11KCC-127	BARI	G101	12AG-247	AZRI
G22	14KCC-102	BARI	G62	11KCC-129	BARI	G102	12AG-248	AZRI
G23	14KCC-103	BARI	G63	11KCC-130	BARI	G103	CM/731/06	NIAB
G24	14KCC-104	BARI	G64	10KCC-101	BARI	G104	CM/736/06	NIAB
G25	14KCC-107	BARI	G65	10KCC-102	BARI	G105	CM/742/06	NIAB
G26	14KCC-108	BARI	G66	10KCC-111	BARI	G106	CM/762/06	NIAB
G27	14KCC-109	BARI	G67	10KCC-112	BARI	G107	CM/771/06	NIAB
G28	14KCC-110	BARI	G68	10KCC-113	BARI	G108	CM/792/06	NIAB
G29	14KCC-111	BARI	G69	10KCC-114	BARI	G109	CM/813/06	NIAB
G30	14KCC-114	BARI	G70	9KCC-160	BARI	G110	FS-4	PRI
G31	14KCC-115	BARI	G71	9KCC-163	BARI	G111	FS-5	PRI
G32	13KCC-101	BARI	G72	9KCC-163	BARI	G112	FS-6	PRI
G33	13KCC-102	BARI	G73	9KCC-164	BARI	G113	FS-7	PRI
G34	13KCC-103	BARI	G74	9KCC-172	BARI	G114	FS-8	PRI
G35	13KCC-105	BARI	G75	8KCC-151	BARI	G115	FS-9	PRI
G36	13KCC-108	BARI	G76	8KCC-152	BARI	G116	FS-10	PRI
G37	13KCC-110	BARI	G77	8KCC-153	BARI	G117	FS-13	PRI
G38	13KCC-111	BARI	G78	8KCC-154	BARI	G118	CM-2008 (C)	NIAB
G39	13KCC-112	BARI	G79	7KCC-154	BARI	G119	TAMMAN (C)	BARI
G40	13KCC-113	BARI	G80	7KCC-156	BARI	G120	NOOR-2013 (C)	NIAB

BARI: Barani Agricultural Research Institute, Chakwal, NIAB: (Nuclear Institute for Agriculture and Biology Faisalabad); AZRI: (Arid Zone Research Institute, Bakkhar); PRI: (Pulses Research Institute, AARI Faisalabad)

### 2.2 Statistical analysis

The statistical analyses were performed by using R software with the “metan” package (Olivoto and Dal’Col Lúcio, 2020).

## 3 Results

### 3.1 Mean Performance, variance components and Likelihood ratio tests (LRT)

**Fig. 2:**
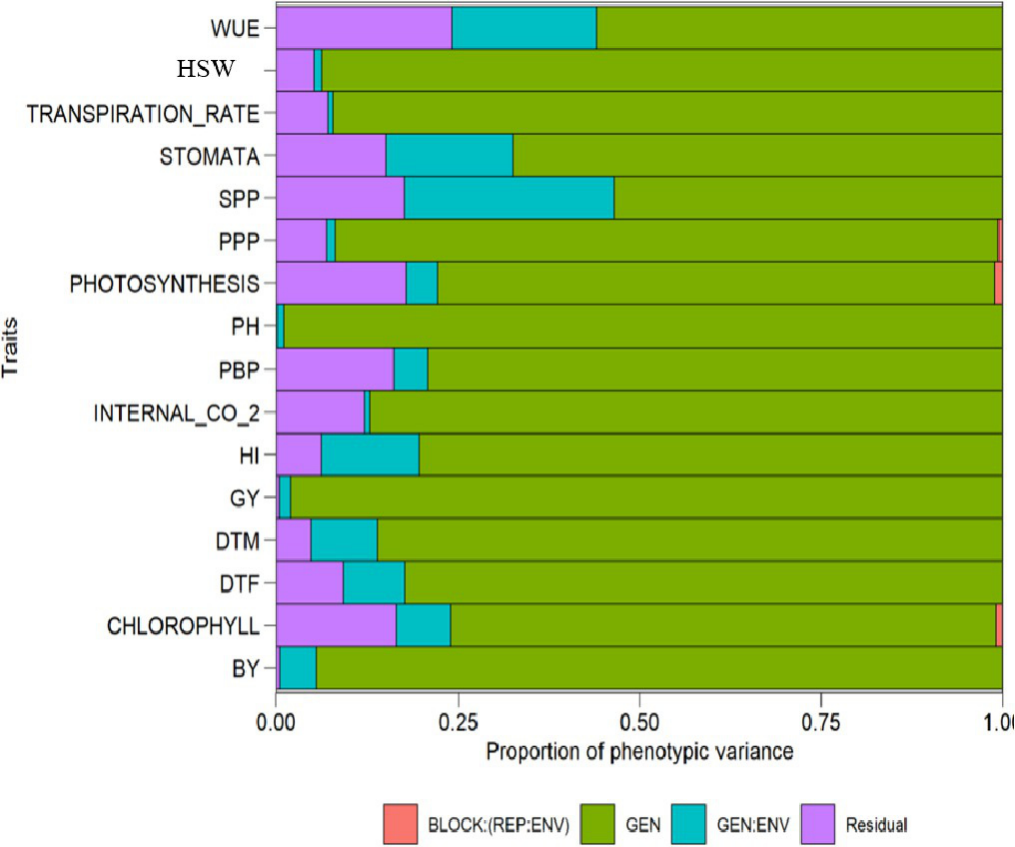
Proportion of the phenotypic variance for 16 Kabuli Chickpea traits assessed in six diverse environments.Where WUE: Water use efficiency, HSW: 100 seed weight, STOMATA: Stomatal conductance, SPP: Number of Seeds per pod, PPP: Number of Pods per plant, Photosynthesis rate, PH: Plant height, PBP: Number of primary branches per plant, INTERNAL_CO_2: Intercellular CO_2_ concentration, HI: Harvest index, GY: Grain yield, DTM: Days to Maturity, DTF: Days to fifty percent flowering, CHLOR: Chlorophyll contents and BY: Biological yield.

The LR test showed significant genotype environment interaction for all traits except for internal CO_2_ concentration (Table 2). The overall grain yield of Kabuli chickpea genotypes ranged from 102 kg ha to 1633 kg/ha while the environments mean yield was 867.50 kg/ha. Others statistical factors such as, mean, standard error of mean (SEM), standard deviation (St dev), 1st and 2nd quartiles for characters in all environments (Table 3). Proximally 82% of the phenotypic variance was due to the genotypic variance. The portion of residual variance was 7.82% and genotype × environment interaction contribution was 9.94% only. For all traits (except for internal CO_2_), the genotypic variance was higher than residual and GEI variance (Fig. 2). High values of broad sense heritability were calculated for all traits under study except for STOMA, seeds per pods and WUE exhibited low heritability. The genotypic selection accuracy (AS) values ranged from 0.94 (SPP) to 0.99 (GY). The highest CVg was recorded for all traits except for the DTF, DTM and internal CO_2_, which showed low CVg Table 4.

**Table 2:** Likelihood ratio test (LRT) for different traits under studied of 120 Kabuli Chickpea Genotypes evaluated in six environments.

Traits	LRT	P-value
GY	632	1.55 × 10^-13^
BY	1170	1.96 × 10^-25^
PH	873	8.70 × 10^-19^
PBP	34.6	4.11 × 10^-9^
SPP	333	2.62 × 10^-74^
HSW	18.3	0.0000186
PPP	12	0.000524
HI	439	2.12 × 10^-97^
DTF	175	4.90 × 10^-40^
DTM	393	1.58 × 10^-87^
PHOTO	24.6	7.15 × 10^-7^
STOMA	231	4.38 × 10^-52^
CHLOR	66.9	2.88 × 10^-16^
TRANS	4.03	0.0446
INT CO_2_	2.71	0.0996
WUE	150	1.45 × 10^-34^

Where GY: Grain yield Kg ha^−1^, BY: Biological yield Kg ha^−1^, PH: Plant height in cm, PBP: Number of primary branches per plant, SPP: No. of seeds per pod, HSW: 100 seed weight, PPP: Number of pods per plant, HI: Harvest index %, DTF: Days to fifty percent flowering, DTM: Days to maturity, PHOTO: Photosynthesis rate, µmoles CO_2_ m^−2^*sec*^-1^, STOMA: Stomatal conductance, Mol H_2_O m^−2^*sec*^-1^, CHLOR: Chlorophyll contents, mg g^−1^ fresh weight, TRANS: Transpiration rate, mMol H_2_O m^−2^*sec*^-1^), INT. CO_2_: Internal CO_2_ concentration, vpm and WUE: Water use efficiency.

**Table 3:** Basic descriptive statistic for various morphological and physiological traits of Kabuli Chickpea genotypes under studied evaluated in six environments.

Traits	Mean	SE Mean	St Dev	Minimum	Q1	Q3	Maximum
PH	72.12	0.28	10.54	41.34	65.82	80.35	93.34
DTF	139.78	0.38	14.51	101.00	139.00	149.00	159.00
DTM	177.82	0.37	14.13	140.00	176.00	187.00	199.00
GY	867.50	0.91	34.45	102.00	80.00	127.00	1633.00
BY	2307.00	2.23	84.70	217.00	187.00	287.00	4157.00
PBP	1.80	0.01	0.53	1.00	1.40	2.00	3.00
SPP	1.57	0.01	0.28	1.00	1.40	1.80	2.00
HSW	22.23	0.10	3.78	14.67	19.48	24.33	34.65
PPP	21.30	0.12	4.72	13.00	18.00	24.00	40.00
HI	44.92	0.16	6.18	21.45	41.00	49.15	65.00
PHOTO	10.40	0.11	3.99	2.00	8.29	13.52	18.93
STOMA	0.22	0.00	0.05	0.14	0.19	0.25	0.37
CHLOR	1.66	0.01	0.26	0.99	1.48	1.81	2.77
TRANS	4.10	0.02	0.82	2.00	3.55	4.63	5.93
INT CO_2_	514.14	1.41	53.40	392.00	475.12	559.00	632.00
WUE	2.68	0.01	0.43	1.08	2.40	2.95	3.87

Where PH: Plant height in cm, DTF: Days to fifty percent flowering, DTM: Days to maturity, GY: Grain yield Kg ha^−1^, BY: Biological yield Kg ha^−1^, PBP: Number of primary branches per plant, SPP: Number of seeds per pod, HSW: 100 seed weight, PPP: Number of pods per plant, HI: Harvest index %, PHOTO: Photosynthesis rate, µmoles CO_2_ m^−2^*sec*^-1^, STOMA: Stomatal conductance, Mol H_2_O m^−2^*sec*^-1^, CHLOR: Chlorophyll contents, mg g^−1^ fresh weight, TRANS: Transpiration rate, mMol H_2_O m^−2^*sec*^-1^), INT. CO_2_: Internal CO_2_ concentration, vpm and WUE: Water use efficiency, S Dev:, Standard deviation, SEM: Standard error of mean, Q1: 1st quartile, Q3: 3rd quartile.

**Table 4:** Deviance analysis, genetic parameters and variance components for 16 Morphological and physiological traits evaluated in 120 Kabuli Chickpea genotypes

Traits	PV	Heritability	GEI R^2^	h^2^mg	AS	r_ge_	CVg	CVr	CV ratio
PH	11.227	0.989	0.009	0.998	0.999	0.848	4.621	0.189	24.452
DTF	8.181	0.824	0.084	0.974	0.987	0.480	1.858	0.619	3.003
DTM	6.716	0.862	0.092	0.978	0.989	0.663	1.353	0.315	4.301
GY	672.144	0.980	0.015	0.997	0.998	0.777	24.520	1.652	14.839
BY	4293.254	0.946	0.049	0.991	0.995	0.902	26.515	1.989	13.329
PBP	0.188	0.792	0.047	0.974	0.987	0.226	21.399	9.645	2.219
SPP	0.078	0.535	0.290	0.895	0.946	0.623	12.994	7.427	1.749
HSW	9.873	0.939	0.010	0.994	0.997	0.166	13.696	3.201	4.279
PPP	17.393	0.914	0.011	0.992	0.996	0.139	18.715	5.145	3.637
HI	30.109	0.804	0.135	0.967	0.983	0.690	10.950	3.015	3.632
PHOTO	1.966	0.768	0.044	0.972	0.986	0.196	11.812	5.686	2.077
STOMA	0.001	0.674	0.176	0.942	0.970	0.539	9.723	4.589	2.119
CHLORO	0.033	0.752	0.076	0.966	0.983	0.316	9.508	4.440	2.141
TRANS	0.243	0.923	0.006	0.993	0.996	0.079	11.549	3.197	3.612
INT CO_2_	234.143	0.872	0.008	0.987	0.994	0.064	2.778	1.032	2.693
WUE	0.075	0.559	0.199	0.913	0.956	0.452	7.665	5.037	1.522

Where PH: Plant height in cm, DTF: Days to fifty percent flowering, DTM: Days to maturity, GY: Grain yield Kg ha^−1^, BY: Biological yield Kg ha^−1^, PBP: Number of primary branches per plant, SPP: Number of seeds per pod, HSW: 100 seed weight, PPP: Number of pods per plant, HI: Harvest index %, PHOTO: Photosynthesis rate, µmoles CO_2_ m^−2^*sec*^-1^, STOMA: Stomatal conductance, Mol H_2_O m^−2^*sec*^-1^, CHLOR: Chlorophyll contents, mg g^−1^ fresh weight, TRANS: Transpiration rate, mMol H_2_O m^−2^*sec*^-1^), INT. CO_2_: Internal CO_2_ concentration, vpm and WUE: Water use efficiency, PV: phenotypic variance, GEI R^2^: GEI coefficient of determination, h^2^mg: heritability of genotypic mean, AS: accuracy of genotype selection, r_ge_, association among genotypic values across environments, CVg: genotypic coefficient of variation, CVr: residual coefficient of variation.

### 3.2 Association analysis

**Fig. 3:**
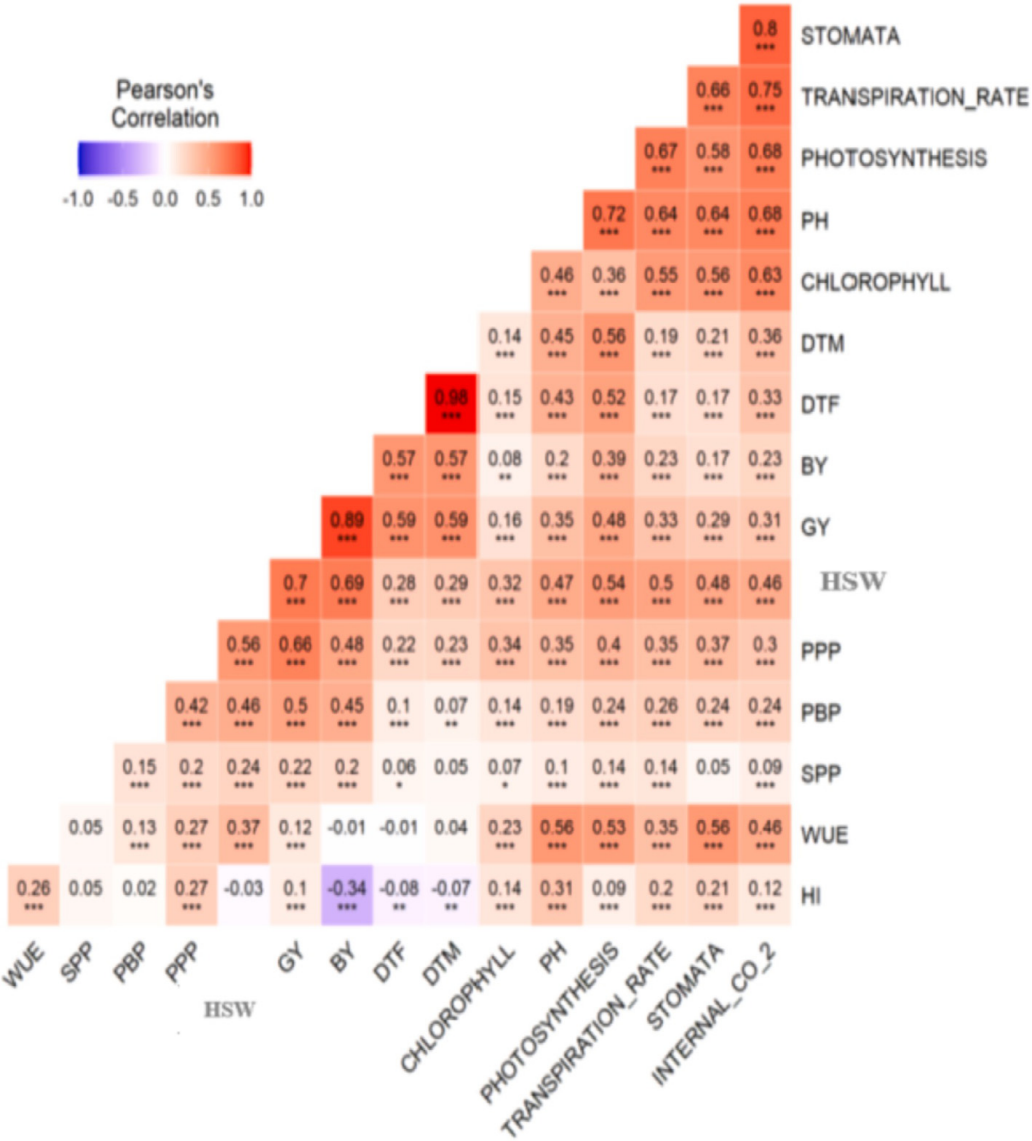
Pearson’s correlation matrix among 16 Kabuli Chickpea traits evaluated in six environments.Where WUE: Water use efficiency, SPP: Number of Seeds per pod, PBP: Number of primary branches per plant, PPP: Number of Pods per plant, HSW: 100 seed weight, GY: Grain yield, BY: Biological yield, DTF: Days to fifty percent flowering, DTM: Days to Maturity, PH: Plant height, STOMATA: Stomatal conductance, INTERNAL_CO_2: Intercellular CO_2_ concentration.

High extent of association was noticed between grain yield and TSW, DTF, DTM and CHLOR contents. Positive and highly significant interactions were found between GY and DTF, PPP and TGW (Fig. 3).

## 4 Genotypes selection based on MTSI and contribution of factors to the MTSI

Nine principal factors were maintained, and the accumulated variance in these factors was 53.85% (Table 5). After proper varimax rotation, mean communality (*h*) was 0.82 signifying that higher ratio of each trait variance was influenced by the factors. The 16 attributes were clustered into the nine different factors as: FA1: (GY and TSW); FA2: (DTF, DTM and CHOLOR); FA3: (PHOTO and WUE); FA4: (TRANS and INT CO_2_), FA5: (PBP); FA6: (BY and HI); F7: (PH and PPP): F8: (STOMA) and F9 (SPP) (Table 6).Table 7.

**Table 5:** Explained variance, Eigenvalues, factorial loadings after varimax rotation and communalities estimated in the factor analysis.

Traits	FA1	FA2	FA3	FA4	FA5	FA6	FA7	FA8	FA9	Cmnlty	Uniq
PH	−0.445	−0.134	−0.258	0.318	0.261	0.204	**−0.474**	−0.005	−0.261	0.787	0.213
DTF	0.003	**0.954**	0.056	−0.031	0.035	0.040	−0.033	−0.030	−0.011	0.920	0.080
DTM	−0.073	**0.945**	0.060	−0.018	0.085	0.075	0.032	−0.010	−0.036	0.918	0.082
GY	**−0.606**	0.066	0.132	−0.024	0.310	−0.316	0.245	−0.013	0.316	0.746	0.254
BY	−0.345	0.018	−0.093	−0.033	0.099	**−0.779**	0.056	−0.115	0.023	0.763	0.237
PBP	−0.037	0.085	0.096	−0.125	**0.897**	−0.181	0.034	0.035	−0.045	0.874	0.126
SPP	−0.097	−0.033	0.008	−0.005	−0.031	0.025	−0.044	−0.098	**0.938**	0.903	0.097
HSW	**−0.886**	0.090	0.085	0.033	−0.057	−0.045	0.112	0.047	0.033	0.823	0.177
PPP	−0.221	−0.021	−0.008	0.050	0.068	−0.013	**0.906**	−0.054	−0.074	0.885	0.115
HI	0.094	−0.087	−0.024	0.075	0.093	**−0.859**	0.005	−0.005	−0.034	0.770	0.230
PHOTO	−0.110	0.065	**0.860**	0.266	0.055	0.022	0.021	−0.106	−0.034	0.843	0.157
STOMA	−0.030	−0.064	−0.054	0.019	0.025	0.087	−0.049	**0.973**	−0.098	0.975	0.025
CHLOR	0.354	**−0.470**	−0.049	0.386	0.070	0.278	−0.051	0.214	−0.121	0.642	0.358
TRANS	−0.060	0.010	0.044	**0.874**	0.036	−0.039	−0.012	−0.029	0.081	0.779	0.221
INT. CO_2_	0.023	−0.121	0.185	**0.707**	−0.374	−0.048	0.041	0.060	−0.131	0.713	0.287
WUE	−0.013	0.060	**0.911**	−0.057	0.032	0.071	0.019	0.032	0.050	0.846	0.154
Eigenvalues	2.21	4.35	0.67	0.60	1.17	1.91	3.56	0.48	1.03	–	–
Variance	6.91	9.07	2.11	1.88	7.33	5.98	11.14	3.02	6.41	–	–
Acc. Var.(%)	6.91	15.98	18.08	19.96	27.30	33.28	44.42	47.44	53.85	–	–

Where PH: Plant height in cm, DTF: Days to fifty percent flowering, DTM: Days to maturity, GY: Grain yield Kg ha^−1^, BY: Biological yield Kg ha^−1^, PBP: Number of primary branches per plant, SPP: Number of seeds per pod, HSW: 100 seed weight, PPP: Number of pods per plant, HI: Harvest index %, PHOTO: Photosynthesis rate, µmoles CO_2_ m^−2^*sec*^-1^, STOMA: Stomatal conductance, Mol H_2_O m^−2^*sec*^-1^, CHLOR: Chlorophyll contents, mg g^−1^ fresh weight, TRANS: Transpiration rate, mMol H_2_O m^−2^*sec*^-1^), INT. CO_2_: Internal CO_2_ concentration, vpm and WUE: Water use efficiency, **FA**, the factor retained, **Bold** values show the traits cluster within each factor, Cmnlty: Communality, Uniq: Uniqueness, Acc. Var. (%) Accumulated variance.

**Table 6:** Selection differential of the WAASBY index for 16 Kabuli Chickpea traits

Factor	Traits	Xo	Xs	SD	SD (%)
FA 1	GY	57.452	63.909	6.457	11.24
	HSW	62.834	68.299	5.465	8.698
FA 2	DTF	68.657	70.282	1.625	2.367
	DTM	66.485	69.734	3.249	4.887
	CHLOR	65.225	64.969	−0.256	−0.393
FA 3	PHOTO	57.702	63.916	6.213	10.768
	WUE	56.264	58.394	2.131	3.787
FA 4	TRANS	78.482	83.326	4.844	6.172
	INT CO_2_	77.538	83.551	6.014	7.756
FA 5	PBP	60.086	71.196	11.11	18.491
FA 6	BY	57.17	60.381	3.211	5.617
	HI	68.622	73.18	4.558	6.643
FA 7	PH	57.868	59.804	1.936	3.346
	PPP	66.42	71.354	4.934	7.428
FA 8	STOMA	59.55	73.427	13.877	23.303
FA 9	SPP	50.556	53.427	2.872	5.375

Where PH: Plant height in cm, DTF: Days to fifty percent flowering, DTM: Days to maturity, GY: Grain yield Kg ha^−1^, BY: Biological yield Kg ha^−1^, PBP: Number of primary branches per plant, SPP: Number of seeds per pod, HSW: 100 seed weight, PPP: Number of pods per plant, HI: Harvest index %, PHOTO: Photosynthesis rate, µmoles CO_2_ m^−2^*sec*^-1^, STOMA: Stomatal conductance, Mol H_2_O m^−2^*sec*^-1^, CHLOR: Chlorophyll contents, mg g^−1^ fresh weight, TRANS: Transpiration rate, mMol H_2_O m^−2^*sec*^-1^), INT. CO_2_: Internal CO_2_ concentration, vpm and WUE: Water use efficiency, Xo: Mean for WAASBY index of the original population, Xs: Mean for WAASBY index of the selected genotypes, SD: Selection Differential.

**Table 7:** Selection Gain (%) for the mean of 16 Kabuli Chickpea traits

VAR	Factor	xo	Xs	SG	SG percent	Sense	Goal
GY	FA 1	104.67	113.61	8.93	8.53	Increase	100
HSW	FA 1	22.23	23.24	1.02	4.57	Increase	100
DTF	FA 2	139.78	138.98	−0.80	−0.57	Decrease	100
DTM	FA 2	177.82	177.19	−0.62	−0.35	Decrease	100
CHLORO	FA 2	1.66	1.63	−0.03	−1.56	Increase	100
PHOTO	FA 3	10.40	10.96	0.55	5.32	Increase	100
WUE	FA 3	2.68	2.72	0.04	1.58	Increase	100
TRANS	FA 4	4.10	4.25	0.15	3.67	Increase	100
INT CO_2_	FA 4	514.14	518.29	4.16	0.81	Increase	100
PBP	FA 5	1.80	2.04	0.23	13.00	Increase	100
BY	FA 6	240.30	268.31	28.01	11.66	Increase	100
HI	FA 6	43.84	44.92	1.97	1.97	Increase	100
PH	FA 7	72.12	71.95	−0.17	−0.23	Decrease	100
PPP	FA 7	21.30	22.79	1.49	6.99	Increase	100
STOMA	FA 8	0.22	0.23	0.01	6.82	Increase	100
SPP	FA 9	1.56	1.57	0.01	0.34	Increase	100

Where PH: Plant height in cm, DTF: Days to fifty percent flowering, DTM: Days to maturity, GY: Grain yield Kg ha^−1^, BY: Biological yield Kg ha^−1^, PBP: Number of primary branches per plant, SPP: Number of seeds per pod, HSW: 100 seed weight, PPP: Number of pods per plant, HI: Harvest index %, PHOTO: Photosynthesis rate, µmoles CO_2_ m^−2^*sec*^-1^, STOMA: Stomatal conductance, Mol H_2_O m^−2^*sec*^-1^, CHLOR: Chlorophyll contents, mg g^−1^ fresh weight, TRANS: Transpiration rate, mMol H_2_O m^−2^*sec*^-1^), INT. CO_2_: Internal CO_2_ concentration, vpm and WUE: Water use efficiency, Xo: Mean for traits of the original population, Xs: Mean for traits of the selected genotypes, SG: Selection gain.

Genotype values for the MTSI presuming 15% selection intensity (Fig-5). Eighteen advanced lines selected were G20, G86, G31, G28, G116, G12, G105, G45, G50, G10, G30, G117, G81, G48, G85, G17, G32, G4, and G37. The MTSI value 8.90 presents the cut point (Fig. 5, red circle). The G37 and G4 genotypes were closer to red circle which possibly will explain desirable characters. Hence, in upcoming research, it would be desirable to explore the performance of the genotypes nearer or closer to the basepoint.

**Fig. 4:**
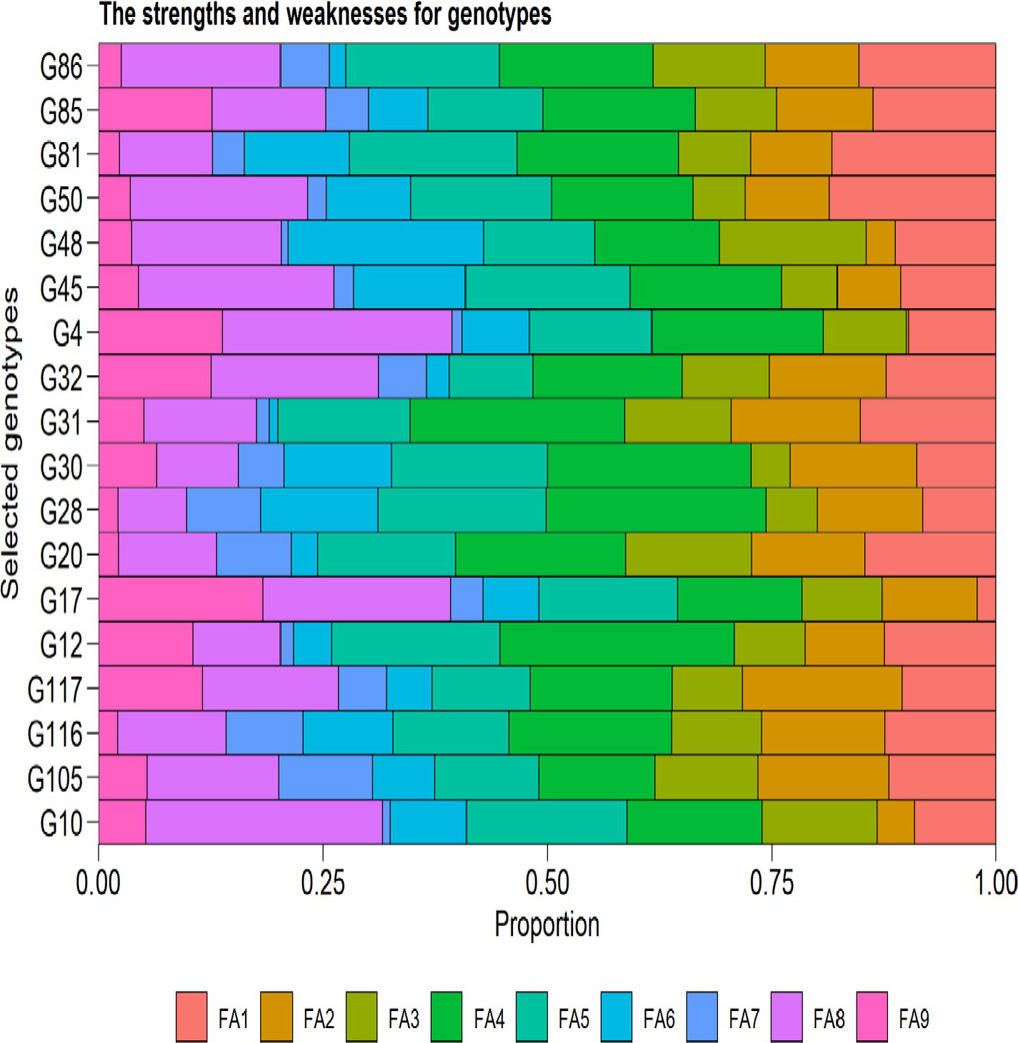
The strengths and weaknesses view of genotypes selected. The y-axis presents the ratio of each factor on the calculated MTSI of the selected genotypes. The minimum the proportions explicated by a factor, the nearer the traits within that factor are to the ideotype. Where G stands for genotypes and FA stands for factor.

The role of factor individually to the MTSI index is used to identify the strengths and weakness of genotypes. The less involvement of a FA, the nearer the characters within that factor are to the ideotype (Fig. 4). For example FA1 (GY and TSW) was the factor with the less contribution to MTSI of G17. Thus, positive gains are desired for GY and TSW. This specifies that this was the high grain yielding genotype out of the superior selected ones (Fig. 4). Further, FA1 was the main causative factor for the MTSI of G50, signifying that this genotype has less production (Fig. 4). The smallest contribution of FA9 was observed for G81, G86, G20, G28 and G116.While for study traits in FA9, higher magnitudes are most required. These genotypes must subsequently have concurrently high magnitude for the traits within that factor. The smallest contributions of FA2 for G30 implies that these advanced lines have high values of DTF, DTM and CHLORO as compared to G11, which has revealed more role for FA2. The less contribution of FA7for G10 (Fig. 4) presented that genotype has a short stature. The selection differential (SD) for the WAASBY index was positive for the traits under present investigation except CHOLOR, suggesting that the technique was more proficient for selection of best performing and most stable advanced lines under diverse environments. The SD for the WAASBY index was 11.2%, 18.49% and 23.30% for GY, PBP and STOMA respectively (Table 6).

**Fig. 5:**
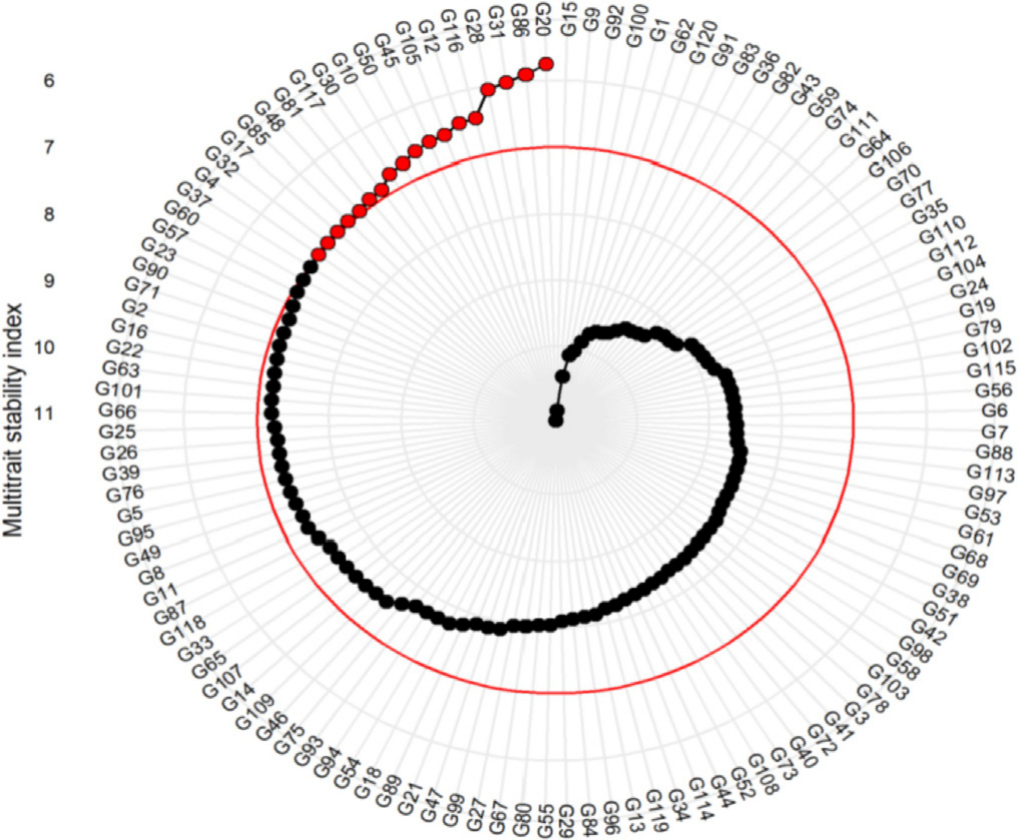
Genotypes selected on the basis of multi trait stability index considering 15 % selection intensity.

## 5 Discussion

Multi Trait Stability Index is useful technique to identify the drought resilient genotypes under water stress environments as more than 90% of chickpea is grown under rainfed environment(Kumar and Abbo, 2001). This approach is valuable to plant breeders as in our study we were able to select genotypes out of 120 genotypes into most stable and high yielding genotypes (G20, G86, G31, G28, G116, G12, G105, G45, G50, G10, G30, G117, G81, G48, G85, G17, G32, G4, and G37). Thus it is recommended that identified traits should be utilized as genitors in hybridization programs for the development of drought tolerant Kabuli Chickpea breeding material (Daryanto et al., 2015, Shah et al., 2020). For all traits (except for intercellular CO_2_), the genotypic variance was higher than residual and GEI variance; as a result, genotypic variance is more considerable constituent of the phenotypic variance (Fig. 2). Therefore, high values of broad sense heritability were calculated for all traits under study except for STOMA, seeds per pods and WUE exhibiting low heritability. This implies that the expected gain from selection would be high if the traits having high heritability values are used as selection criteria in chickpea breeding program. Our results are in line with earlier studies where they reported highest heritability for pods per plant, days to 50% flowering, hundred seed weight, harvest index and grain yield per plant. (Yücel et al., 2006, Arora and Kumar, 2018, Banik et al., 2018, Hagos et al., 2018, Sharifi et al., 2018, Kumar et al., 2019). Similarly, genetic diversity of 25 chickpea genotypes was studied using multivariate technique. The results showed that first three principal components depicted 69.69% variations. The three factors were phenological traits (33.69%), morphological traits (20.82%) and yield components (15.19%) (Sharifi et al., 2018). Based upon our results and as reported in the previous studies outcomes from these works can be used in the breeding strategies for the classification of diversity among genotypes. Furthermore, it can also be used for yield improvement through hybridization programs. Our results reported high extent of association between grain yield and TSW, DTF, DTM and CHLOR contents. Positive and highly significant interactions were found between GY and DTF, PPP and TGW indicating that these characters had good relationship with grain yield in Kabuli chickpea, therefore, were important characters for bringing genetic improvement in grain yield. Plant breeders can also focus their attention on the traits having strong correlation with grain yield to develop better genotypes of Kabuli chickpea. A strong association between GY with TSW, DTF, DTF and PPP has been reported in previous work (Noor et al., 2003, Arshad et al., 2004, Kumar et al., 2019).

Our selection based on the multi traits may assemble genotypes with a superior adaptability across prevailing weather conditions of immense significance for hybridization programs. Recently it has been proposed that the WAASB (Weighted Average of Absolute Scores) index could be good indicator for selection of superior genotype on the base of multi-traits in multi-environment (Olivoto et al., 2019a, Olivoto et al., 2019b). Principally, this index is computed by using the single value decomposition of the BLUPs matrix (Best Non-Impartial Linear Forecast) for the GEI sound effects produced by an LMM. The genotypes with the lower WAASB index values have the wider stability on the basis of studied traits in the evaluated environments. So the technique used by Olivoto *et al.* (2019) permitted for selection of the better advanced lines in the six evaluated environments (RBFI, RBLI, RBDS, BRFC, FRFC, ARFC) on the basis of data collected from a set of sixteen traits. Fig. 5 shows the ranking of genotypes for the MTSI presuming 15% selection intensity, eighteen genotypes were selected *viz*: G20, G86, G31, G28, G116, G12, G105, G45, G50, G10, G30, G117, G81, G48, G85, G17, G32, G4, and G37 as the most stable out of 120 genotypes in present research work. The MTSI of 9.90 serve as the cut point (Fig. 5, red circle) considering the selection intensity. The G37 and G4 genotype were in close proximity to this red circle and may possibly possess remarkable characters. Therefore, in further research, it would be attractive to explore the performance of the genotypes near and close to the base cut point (Olivoto et al., 2019a, Olivoto et al., 2019b).

The selection accomplished in Fig. 3 served as a source to estimate genetic attributes for each analyzed trait considering a selection intensity of 15 (Table-5). For all the investigated attributes, the mean of the genotypes selected (Xs) was greater than the mean of original population (Xo), comprised of all 120 genotypes except for chlorophyll which mean this attribute was more affected by environmental conditions as compared to others evaluated traits. The extent of this percentage increase varied as a function of the investigated trait and encouraged stress in the genotypes (Table 6). The SD was from 2.37% (DTF) to 23.30% (STOMA) between the six environments evaluated and the various traits under study, which represents the prospect of achieving gain with selection on all traits recorded (Table 6).

There is very meager information available in literature for selection of genotypes on the basis of multi traits multi environment trials. So according to present climate change scenario, the chickpea breeder should focus on this aspect for selection of superior genotypes which perform better under diverse environmental conditions as the genotypes affected generally by the significant GEI that happens in the majority of the crops including chickpea. In the present era to ensure the food security, agriculture sector must fulfill the demands for food in the changing climate scenario, while mitigating the unfavorable impacts of agriculture on the weather conditions. The solution to achieving this valuable task is to develop breeding material having comprehensive genetic variation at different plant growth stages in reaction to the various abiotic and biotic stresses (Bailey-Serres et al., 2019, Zuffo et al., 2020). So the most imperative aim is to assess the genotypes under various environmental conditions and to choose those better genotypes which perform superior under changing climatic condition from one region to others. The incorporation of MTSI research permitted us to express scientific solutions to stress experiments. In the present evaluation, genetic stability was computed by using MTSI in six different environments presenting the efficacy of this technique proposed by Olivoto *et al.* (2019). Drought stress generally reduces different plants growth stages and ultimately grain yield in a broad sense, by altering physiological and morphological changes (Shah et al., 2020). To cope and understand these variations in plants when assessing a comparatively a greater number of genotypes, as well as several stresses concurrently, it is essential to utilize suitable techniques to achieve desirable goals. Techniques such as principal component analysis (Giordani et al., 2019), BLUP (Olivoto et al., 2017), AMMI (additive main effects and multiplicative interaction) (Nowosad et al., 2016, Bocianowski et al., 2018, Olivoto et al., 2019a, Olivoto et al., 2019b), the combination of BLUP and AMMI, and MTSI (Olivoto et al., 2019a, Olivoto et al., 2019b). have been used but the information on MTSI in chickpea is very meager. According to the computed MTSI for multiple environments, genotypes G20, G86, G31, G28, G116, G12, G105, G45, G50, G10, G30, G117, G81, G48, G85, G17, G32, G4, and G37 selected as the most stable and high yielding among the 120 genotypes in present research work. The genotypes selected from the present research are best to be utilized in breeding program for development of superior genotypes to perform better in diverse environmental conditions. The high selection gains explained that character’s variation is mainly owing to genetic makeup and hence probably to be incorporated to potential future fillial generations through breeding techniques. The superior genotypes can be utilized as genitors in future hybridization plans for the development of breeding material of Kabuli chickpea resilient to abiotic stresses.

## 6 Conclusion

Drought tolerance evaluation in six environments led us to conclude that MTSI could be used to select superior chickpea genotypes with improved yield traits. Genotypes were categorized into groups based on their performance under set of variable environments. We were able to identify genotypes that showed differential response under irrigated and water stress environments while some performed well under both set of environments. The MTSI is estimated based on the genotype ideotype distance projected with values of factor analysis. Accordingly, genotypes G20, G86, G31, G28, G116, G12, G105, G45, G50, G10, G30, G117, G81, G48, G85, G17, G32, G4, and G37 selected as the most stable and high yielding among the 120 genotypes. The MTSI technique presented the selection of most stable genotypes for the traits to be increased with positive selection differentials and negative selection differential for attributes that required to be reduced. The MTSI may be valuable for the plant breeders for the selection of genotypes for MPS based on multiple traits as it gives a strong and simple to understand process of selection. Furthermore in future identified materials can be used as genitors in breeding programs with the aim to have offspring with higher yield and resistance to abiotic stress.

We recommend that breeder should apply MTSI to identify high yielding stable drought tolerant genotypes prior to testing them under multiple environments which is ultimately required for approving variety in different environments. This technique will be best for the countries where resources are limited as it will save time and cost. This study also provides useful information to policy makers and provides directions for the development of stable drought tolerant resilient chickpea cultivars in the water stress environments.

## Declaration of Competing Interest

The authors declare that they have no known competing financial interests or personal relationships that could have appeared to influence the work reported in this paper.

## References

[b0005] Abbas G., Ahmad S., Ahmad A., Nasim W., Fatima Z., Hussain S., Rehman M.H.u., Khan M.A., Hasanuzzaman M., Fahad S., Boote K.J., Hoogenboom G. (2017). Quantification the impacts of climate change and crop management on phenology of maize-based cropping system in punjab, pakistan. Agricultural and Forest Meteorology.

[b0010] Ahmad F., Gaur P., Croser J. (2005). Chickpea (cicer arietinum l.). Genetic resources, chromosome engineering, and crop improvement-grain legumes.

[b0015] Ahmad S., Abbas G., Ahmed M., Fatima Z., Anjum M.A., Rasul G., Khan M.A., Hoogenboom G. (2019). Climate warming and management impact on the change of phenology of the rice-wheat cropping system in punjab, pakistan. Field Crops Research.

[b0020] Ahmad S., Abbas Q., Abbas G., Fatima Z., Atique Ur R., Naz S., Younis H., Khan R.J., Nasim W., Habib Ur Rehman M., Ahmad A., Rasul G., Khan M.A., Hasanuzzaman M. (2017). Quantification of climate warming and crop management impacts on cotton phenology. Plants (Basel).

[b0025] Ahmed K., Shabbir G., Ahmed M., Shah K.N. (2020). Phenotyping for drought resistance in bread wheat using physiological and biochemical traits. Science of The Total Environment.

[b0030] Ahmed, M., 2020. Introduction to modern climate change. Andrew e. Dessler: Cambridge university press, 2011, 252 pp, isbn-10: 0521173159. Science of The Total Environment, 734: 139397. Available from http://www.sciencedirect.com/science/article/pii/S0048969720329144. DOI 10.1016/j.scitotenv.2020.139397.

[b0035] Ali M.G.M., Ibrahim M.M., El Baroudy A., Fullen M., Omar E.-S.-H., Ding Z., Kheir A.M.S. (2020). Climate change impact and adaptation on wheat yield, water use and water use efficiency at north nile delta. Frontiers of Earth Science.

[b0040] Amiri, S., H. Eyni-Nargeseh, S. Rahimi-Moghaddam and K. Azizi, 2021. Water use efficiency of chickpea agro-ecosystems will be boosted by positive effects of co2 and using suitable genotype × environment × management under climate change conditions. Agricultural Water Management, 252: 106928. Available from https://www.sciencedirect.com/science/article/pii/S0378377421001931. DOI 10.1016/j.agwat.2021.106928.

[b0045] Araus J.L., Slafer G.A., Reynolds M.P., Royo C. (2002). Plant breeding and drought in c3 cereals: What should we breed for? Ann Bot, 89 Spec No(7): 925–940. DOI.

[b0050] Arif A., Parveen N., Waheed M.Q., Atif R.M., Waqar I., Shah T.M. (2021). A comparative study for assessing the drought-tolerance of chickpea under varying natural growth environments. Frontiers Plant Science.

[b0055] Arora R., Kumar K. (2018). Genetic variability studies for yield contributing traits in kabuli chickpea (cicer arietinum l.). Journal of Pharmacognosy and Phytochemistry.

[b0060] Arshad M., Bakhsh A., Ghafoor A. (2004). Path coefficient analysis in chickpea (cicer arietinum l.) under rainfed conditions. Pakistan Journal of Botany.

[b0065] Azam M., Iqba M., Hossain M., Hossain M. (2020). Stability investigation and genotype ã environment association in chickpea genotypes utilizing ammi and gge biplot model. Genetics and Molecular Research.

[b0070] Bailey-Serres J., Parker J.E., Ainsworth E.A., Oldroyd G.E.D., Schroeder J.I. (2019). Genetic strategies for improving crop yields. Nature.

[b0075] Banik M., Deore G.N., Mandal A.K., Mhase L.B. (2018). Genetic variability and heritability studies in chickpea (cicer arietinum l.). Current Journal of Applied Science and Technology.

[b0080] Barnabás, B., K. Jäger and A. Fehér, 2008. The effect of drought and heat stress on reproductive processes in cereals. Plant, Cell & Environment, 31(1): 11-38. Available from https://onlinelibrary.wiley.com/doi/abs/10.1111/j.1365-3040.2007.01727.x. DOI 10.1111/j.1365-3040.2007.01727.x. 10.1111/j.1365-3040.2007.01727.x 17971069

[b0085] Bocianowski J., Niemann J., Nowosad K. (2018). Genotype-by-environment interaction for seed quality traits in interspecific cross-derived brassica lines using additive main effects and multiplicative interaction model. Euphytica.

[b0090] Caloiero T., Coscarelli R., Gaudio R. (2019). Spatial and temporal variability of daily precipitation concentration in the sardinia region (italy). International Journal of Climatology.

[b0095] Chandora R., Gayacharan N.S., Malhotra N., gains, M. Singh, (2020). Chickpea: Crop wild relatives for enhancing genetic.

[b0100] Chaturvedi S., Jha S., Singh N., Gaur P., Varshney R. (2018). Technological and policy intervention for increasing chickpea production in india. Pulse India.

[b0105] Chibbar R.N., Ambigaipalan P., Hoover R. (2010). Molecular diversity in pulse seed starch and complex carbohydrates and its role in human nutrition and health. Cereal chemistry.

[b0110] Cohen, I., S.I. Zandalinas, C. Huck, F.B. Fritschi and R. Mittler, 2021. Meta-analysis of drought and heat stress combination impact on crop yield and yield components. Physiologia Plantarum, 171(1): 66-76. Available from https://onlinelibrary.wiley.com/doi/abs/10.1111/ppl.13203. DOI 10.1111/ppl.13203. 10.1111/ppl.13203 32880977

[b0115] Daryanto S., Wang L., Jacinthe P.A., Hui D. (2015). Global synthesis of drought effects on food legume production. PLOS ONE.

[b0120] Devasirvatham V., Tan D.K. (2018). Impact of high temperature and drought stresses on chickpea production. Agronomy.

[b0125] Ding, Z., E.F. Ali, A.M. Elmahdy, K.E. Ragab, M.F. Seleiman and A.M.S. Kheir, 2021. Modeling the combined impacts of deficit irrigation, rising temperature and compost application on wheat yield and water productivity. Agricultural Water Management, 244: 106626. Available from https://www.sciencedirect.com/science/article/pii/S0378377420321739. DOI 10.1016/j.agwat.2020.106626.

[b0130] Dixit G., Srivastava A., Singh N. (2019). Marching towards self-sufficiency in chickpea. Current Science.

[b0135] El Sayed, M.A.A., A.M.S. Kheir, F.A. Hussein, E.F. Ali, M.E. Selim, A. Majrashi and E.A.Z. El Shamey, 2021. Developing new lines of japonica rice for higher quality and yield under arid conditions. PeerJ, 9: e11592. Available from DOI: 10.7717/peerj.11592. DOI 10.7717/peerj.11592. 10.7717/peerj.11592 PMC8210806 34178464

[b0140] Erdemci I. (2018). Investigation of genotype× environment interaction in chickpea genotypes using ammi and gge biplot analysis. Turkish Journal Of Field Crops.

[b0145] FAOSTAT, 2019. Food and agriculture organization statistics database. Retrieved march 21, 2020, from http://www.Fao.Org/faostat/en/#data/qc.

[b0150] Fatima, Z., M. Ahmed, M. Hussain, G. Abbas, S. Ul-Allah, S. Ahmad, N. Ahmed, M.A. Ali, G. Sarwar, E.u. Haque, P. Iqbal and S. Hussain, 2020. The fingerprints of climate warming on cereal crops phenology and adaptation options. Scientific Reports, 10(1): 18013. Available from DOI: 10.1038/s41598-020-74740-3. DOI 10.1038/s41598-020-74740-3. 10.1038/s41598-020-74740-3 PMC7581754 33093541

[b0155] Fatima, Z., R. Atique ur, G. Abbas, P. Iqbal, I. Zakir, M.A. Khan, G.M. Kamal, M. Ahmed and S. Ahmad, 2020. Quantification of climate warming and crop management impacts on phenology of pulses-based cropping systems. International Journal of Plant Production. Available from DOI: 10.1007/s42106-020-00112-6. DOI 10.1007/s42106-020-00112-6.

[b0160] Garg R., Bhattacharjee A., Jain M. (2015). Genome-scale transcriptomic insights into molecular aspects of abiotic stress responses in chickpea. Plant Molecular Biology Reporter.

[b0165] Gaur P.M., Krishnamurthy L., Kashiwagi J. (2008). Improving drought-avoidance root traits in chickpea (cicer arietinum l.)-current status of research at icrisat. Plant Production Science.

[b0170] Gaur, P.M., S. Tripathi, C.L. Gowda, G. Ranga Rao, H. Sharma, S. Pande and M. Sharma, 2010. Chickpea seed production manual.

[b0175] Giordani W., Gonçalves L.S.A., Moraes L.A.C., Ferreira L.C., Neumaier N., Farias J.R.B., Nepomuceno A.L., Oliveira M.C.N.d., Henning L. (2019). Identification of agronomical and morphological traits contributing to drought stress tolerance in soybean. Australian Journal of Crop Science.

[b0180] Hagos A.A., Desalegn T., Belay T. (2018). Genetic variability, correlation and path analysis for quantitative traits of seed yield, and yield components in chickpea (cicer arietinum l.) at maichew, northern ethiopia. African Journal of Plant Science.

[b0185] IPCC, 2014. Climate change 2014: Impacts, adaptation, and vulnerability. Part a: Global and sectoral aspects. Contribution of working group ii to the fifth assessment report of the intergovernmental panel on climate change [field, c.B., v.R. Barros, d.J. Dokken, k.J. Mach, m.D. Mastrandrea, t.E. Bilir, m. Chatterjee, k.L. Ebi, y.O. Estrada, r.C. Genova, b. Girma, e.S. Kissel, a.N. Levy, s. Maccracken, p.R. Mastrandrea, and l.L. White (eds.)]. Cambridge, United Kingdom and New York, NY, USA: Cambridge University Press.

[b0190] Jabbari, M., B.A. Fakheri, R. Aghnoum, R. Darvishzadeh, N. Mahdi Nezhad, R. Ataei, Z. Koochakpour and M. Razi, 2021. Association analysis of physiological traits in spring barley (hordeum vulgare l.) under water-deficit conditions. Food Science & Nutrition, 9(3): 1761-1779. Available from https://onlinelibrary.wiley.com/doi/abs/10.1002/fsn3.2161. DOI 10.1002/fsn3.2161. 10.1002/fsn3.2161 PMC7958556 33747487

[b0195] Jamro, S., G.H. Dars, K. Ansari and N.Y. Krakauer, 2019. Spatio-temporal variability of drought in pakistan using standardized precipitation evapotranspiration index. Applied Sciences, 9(21): 4588. Available from https://www.mdpi.com/2076-3417/9/21/4588.

[b0200] Jha U.C., Chaturvedi S.K., Bohra A., Basu P.S., Khan M.S., Barh D., Varshney R. (2014). Abiotic stresses, constraints and improvement strategies in chickpea. Plant Breeding.

[b0205] Kadiyala M., Kumara Charyulu D., Nedumaran S., Shyam M.D., Gumma M., Bantilan M. (2016). Agronomic management options for sustaining chickpea yield under climate change scenario. Journal of Agrometeorology.

[b0210] Kheir, A.M.S., E.F. Ali, Z. He, O.A.M. Ali, T. Feike, M.M. Kamara, M. Ahmed, M.A. Eissa, A.E. Fahmy and Z. Ding, 2021. Recycling of sugar crop disposal to boost the adaptation of canola (brassica napus l.) to abiotic stress through different climate zones. Journal of Environmental Management, 281: 111881. Available from http://www.sciencedirect.com/science/article/pii/S0301479720318065. DOI 10.1016/j.jenvman.2020.111881. 10.1016/j.jenvman.2020.111881 33401121

[b0215] Korres, N.E., J.K. Norsworthy, P. Tehranchian, T.K. Gitsopoulos, D.A. Loka, D.M. Oosterhuis, D.R. Gealy, S.R. Moss, N.R. Burgos, M.R. Miller and M. Palhano, 2016. Cultivars to face climate change effects on crops and weeds: A review. Agron. Sustain. Dev., 36(1): 12. Available from DOI: 10.1007/s13593-016-0350-5. DOI 10.1007/s13593-016-0350-5.

[b0220] Kumar J., Abbo S. (2001). Advances in agronomy.

[b0225] Kumar S., Suresh B., Kumar A., Lavanya G. (2019). Genetic variability, correlation and path coefficient analysis in chickpea (cicer arietinum l.) for yield and its component traits. Int. J. Curr. Microbiol. App. Sci.

[b0230] Kumar, T., N. Tiwari, C. Bharadwaj, A. Sarker, S.P.R. Pappula, S. Singh and M. Singh, 2020. Identification of allelic variation in drought responsive dehydrin gene based on sequence similarity in chickpea (cicer arietinum l.). Frontiers in Genetics, 11(1579). Available from https://www.frontiersin.org/article/10.3389/fgene.2020.584527. DOI 10.3389/fgene.2020.584527. 10.3389/fgene.2020.584527 PMC7767992 33381148

[b0235] Liu L.H., Hung T.V., Bennett L. (2008). Extraction and characterization of chickpea (cicer arietinum) albumin and globulin. Journal of food science.

[b0240] Makonya, G.M., J.B.O. Ogola, A.M. Muasya, O. Crespo, S. Maseko, A.J. Valentine, C.-O. Ottosen, E. Rosenqvist and S.B.M. Chimphango, 2020. Intermittent moisture supply induces drought priming responses in some heat-tolerant chickpea genotypes. Crop Science, 60(5): 2527-2542. Available from https://acsess.onlinelibrary.wiley.com/doi/abs/10.1002/csc2.20228. DOI 10.1002/csc2.20228.

[b0245] Maqbool, M.A., M. Aslam and H. Ali, 2017. Breeding for improved drought tolerance in chickpea (cicer arietinum l.). Plant Breeding, 136(3): 300-318. Available from https://onlinelibrary.wiley.com/doi/abs/10.1111/pbr.12477. DOI 10.1111/pbr.12477.

[b0250] Massawe, F.J., S. Mayes, A. Cheng, H.H. Chai, P. Cleasby, R. Symonds, W.K. Ho, A. Siise, Q.N. Wong, P. Kendabie, Y. Yanusa, N. Jamalluddin, A. Singh, R. Azman and S.N. Azam-Ali, 2015. The potential for underutilised crops to improve food security in the face of climate change. Procedia Environmental Sciences, 29: 140-141. Available from http://www.sciencedirect.com/science/article/pii/S1878029615004661. DOI http://dx.doi.org/10.1016/j.proenv.2015.07.228.

[b0255] Mengistu, A.G., W.A. Tesfuhuney, Y.E. Woyessa and L.D. van Rensburg, 2020. Analysis of the spatio-temporal variability of precipitation and drought intensity in an arid catchment in south africa. Climate, 8(6): 70. Available from https://www.mdpi.com/2225-1154/8/6/70.

[b0260] Mohammadi, R., M. Armion, E. Zadhasan, M.M. Ahmadi and A. Amri, 2017. The use of ammi model for interpreting genotype × environment interaction in durum wheat. Experimental Agriculture, 54(5): 670-683. Available from https://www.cambridge.org/core/article/use-of-ammi-model-for-interpreting-genotype-environment-interaction-in-durum-wheat/9874315F9BE9FD64FC8BDF3630C65722. DOI 10.1017/S0014479717000308.

[b0265] Murungweni, C., M.T. Van Wijk, E.M.A. Smaling and K.E. Giller, 2016. Climate-smart crop production in semi-arid areas through increased knowledge of varieties, environment and management factors. Nutr Cycl Agroecosyst, 105(3): 183-197. Available from DOI: 10.1007/s10705-015-9695-4. DOI 10.1007/s10705-015-9695-4.

[b0270] Najan B., Patil J., Gethe R., Kadam D. (2018). Heterosis and combining ability analysis for yield and yield contributing characters in chickpea (kabuli). Trends in Biosciences.

[b0275] Nicholson, S.E., C. Funk and A.H. Fink, 2018. Rainfall over the african continent from the 19th through the 21st century. Global and Planetary Change, 165: 114-127. Available from http://www.sciencedirect.com/science/article/pii/S0921818117302783. DOI 10.1016/j.gloplacha.2017.12.014.

[b0280] Noor, F., M. Ashaf and A. Ghafoor, 2003. Path analysis and relationship among quantitative traits in chickpea (cicer arietinum l.). Pak. J. Biol. Sci, 6(6): 551-555.

[b0285] Nowosad, K., A. Liersch, W. Popławska and J. Bocianowski, 2016. Genotype by environment interaction for seed yield in rapeseed (brassica napus l.) using additive main effects and multiplicative interaction model. Euphytica, 208(1): 187-194. Available from DOI: 10.1007/s10681-015-1620-z. DOI 10.1007/s10681-015-1620-z.

[b0290] Olivoto, T. and A. Dal’Col Lúcio, 2020. Metan: An r package for multi-environment trial analysis. bioRxiv: 2020.2001.2014.906750. Available from https://www.biorxiv.org/content/biorxiv/early/2020/01/14/2020.01.14.906750.full.pdf. DOI 10.1101/2020.01.14.906750.

[b0295] Olivoto, T., A.D.C. Lúcio, J.A.G. da Silva, V.S. Marchioro, V.Q. de Souza and E. Jost, 2019. Mean performance and stability in multi-environment trials i: Combining features of ammi and blup techniques. Agronomy Journal, 111(6): 2949-2960. Available from https://acsess.onlinelibrary.wiley.com/doi/abs/10.2134/agronj2019.03.0220. DOI https://doi.org/10.2134/agronj2019.03.0220.

[b0300] Olivoto, T., A.D.C. Lúcio, J.A.G. da Silva, B.G. Sari and M.I. Diel, 2019. Mean performance and stability in multi-environment trials ii: Selection based on multiple traits. Agronomy Journal, 111(6): 2961-2969. Available from https://acsess.onlinelibrary.wiley.com/doi/abs/10.2134/agronj2019.03.0221. DOI https://doi.org/10.2134/agronj2019.03.0221.

[b0305] Olivoto T., Nardino M., Carvalho I., Follmann D., Ferrari M., Szareski V., V.d. Souza, (2017). Reml/blup and sequential path analysis in estimating genotypic values and interrelationships among simple maize grain yield-related traits. Genetics and Molecular Research.

[b0310] Purushothaman, R., H.D. Upadhyaya, P.M. Gaur, C.L.L. Gowda and L. Krishnamurthy, 2014. Kabuli and desi chickpeas differ in their requirement for reproductive duration. Field Crops Research, 163: 24-31. Available from https://www.sciencedirect.com/science/article/pii/S0378429014001002. DOI https://doi.org/10.1016/j.fcr.2014.04.006.

[b0315] Rani A., Devi P., Jha U.C., Sharma K.D., Siddique K.H., Nayyar H. (2020). Developing climate-resilient chickpea involving physiological and molecular approaches with a focus on temperature and drought stresses. Frontiers in plant science.

[b0320] Schneider S., Semenov S., Patwardhan A., Burton I., Magadza C., Oppenheimer M., Pittock A., Rahman A., Smith J., Suarez A. (2007).

[b0325] Shah, T.M., M. Imran, B.M. Atta, M.Y. Ashraf, A. Hameed, I. Waqar, M. Shafiq, K. Hussain, M. Naveed, M. Aslam and M.A. Maqbool, 2020. Selection and screening of drought tolerant high yielding chickpea genotypes based on physio-biochemical indices and multi-environmental yield trials. BMC Plant Biology, 20(1): 171. Available from https://doi.org/10.1186/s12870-020-02381-9. DOI 10.1186/s12870-020-02381-9. 10.1186/s12870-020-02381-9 PMC7164285 32303179

[b0330] Sharifi, P., H. Astereki and M. Pouresmael, 2018. Evaluation of variations in chickpea (cicer arietinum l.) yield and yield components by multivariate technique. Annals of Agrarian Science, 16(2): 136-142. Available from https://www.sciencedirect.com/science/article/pii/S1512188717300040. DOI https://doi.org/10.1016/j.aasci.2018.02.003.

[b0335] Singh Mohar, Malhotra Nikhil, Singh Kuldeep (2021). Broadening the genetic base of cultivated chickpea following introgression of wild cicer species-progress, constraints and prospects. Genetic Resources and Crop Evolution. Available from.

[b0340] Talchabhadel, R., R. Karki, B.R. Thapa, M. Maharjan and B. Parajuli, 2018. Spatio-temporal variability of extreme precipitation in nepal. International Journal of Climatology, 38(11): 4296-4313. Available from https://rmets.onlinelibrary.wiley.com/doi/abs/10.1002/joc.5669. DOI https://doi.org/10.1002/joc.5669.

[b0345] Thomas, R.J., 2008. Opportunities to reduce the vulnerability of dryland farmers in central and west asia and north africa to climate change. Agriculture, Ecosystems & Environment, 126(1–2): 36-45. Available from http://www.sciencedirect.com/science/article/pii/S0167880908000212. DOI http://dx.doi.org/10.1016/j.agee.2008.01.011.

[b0350] Thudi, M., H.D. Upadhyaya, A. Rathore, P.M. Gaur, L. Krishnamurthy, M. Roorkiwal, S.N. Nayak, S.K. Chaturvedi, P.S. Basu and N. Gangarao, 2014. Genetic dissection of drought and heat tolerance in chickpea through genome-wide and candidate gene-based association mapping approaches. Plos one, 9(5): e96758. 10.1371/journal.pone.0096758 PMC4011848 24801366

[b0355] Tripathi, A., D.K. Tripathi, D.K. Chauhan, N. Kumar and G.S. Singh, 2016. Paradigms of climate change impacts on some major food sources of the world: A review on current knowledge and future prospects. Agriculture, Ecosystems & Environment, 216: 356-373. Available from https://www.sciencedirect.com/science/article/pii/S0167880915300992. DOI https://doi.org/10.1016/j.agee.2015.09.034.

[b0360] Vadez V., Hajjarpoor A., Korbu L.B., Alimagham M., Pushpavalli R., Ramirez M.L., Kashiwagi J., Kholova J., Turner N.C., Sadras V.O., Sadras V.O., Calderini D.F. (2021). Crop physiology case histories for major crops.

[b0365] Vergni, L. and F. Todisco, 2011. Spatio-temporal variability of precipitation, temperature and agricultural drought indices in central italy. Agricultural and Forest Meteorology, 151(3): 301-313. Available from https://www.sciencedirect.com/science/article/pii/S0168192310003060. DOI https://doi.org/10.1016/j.agrformet.2010.11.005.

[b0370] Vinocur Basia, Altman Arie (2005). Recent advances in engineering plant tolerance to abiotic stress: Achievements and limitations. Current opinion in biotechnology.

[b0375] Voosen, P., 2021. Global temperatures in 2020 tied record highs. American Association for the Advancement of Science. 10.1126/science.371.6527.334 33479133

[b0380] Vurukonda, S.S.K.P., S. Vardharajula, M. Shrivastava and A. SkZ, 2016. Enhancement of drought stress tolerance in crops by plant growth promoting rhizobacteria. Microbiological Research, 184: 13-24. Available from https://www.sciencedirect.com/science/article/pii/S0944501315300380. DOI https://doi.org/10.1016/j.micres.2015.12.003. 10.1016/j.micres.2015.12.003 26856449

[b0385] Wang J.Y., Turner N.C., Liu Y.X., Siddique K.H.M., Xiong Y.C. (2017). Effects of drought stress on morphological, physiological and biochemical characteristics of wheat species differing in ploidy level. Functional plant biology : FPB.

[b0390] Wang Xiansheng, Gao Wenrui, Zhang Jusong, Zhang Hua, Li Jiangui, He Xiaoling, Ma Hao (2010). Subunit, amino acid composition and in vitro digestibility of protein isolates from chinese kabuli and desi chickpea (cicer arietinum l.) cultivars. Food Research International.

[b0395] Waqas, M.A., X. Wang, S.A. Zafar, M.A. Noor, H.A. Hussain, M. Azher Nawaz and M. Farooq, 2021. Thermal stresses in maize: Effects and management strategies. Plants, 10(2): 293. Available from https://www.mdpi.com/2223-7747/10/2/293. 10.3390/plants10020293 PMC7913793 33557079

[b0400] Yang, W., G. Feng, A. Adeli, H. Tewolde and Z. Qu, 2021. Simulated long-term effect of wheat cover crop on soil nitrogen losses from no-till corn-soybean rotation under different rainfall patterns. Journal of Cleaner Production, 280: 124255. Available from https://www.sciencedirect.com/science/article/pii/S0959652620343006. DOI https://doi.org/10.1016/j.jclepro.2020.124255.

[b0405] Yücel D.Ö., Anlarsal A.E., Yücel C. (2006). Genetic variability, correlation and path analysis of yield, and yield components in chickpea (cicer arietinum l.). Turkish Journal of Agriculture and Forestry.

[b0410] Zampieri, M., C.J. Weissteiner, B. Grizzetti, A. Toreti, M. van den Berg and F. Dentener, 2020. Estimating resilience of crop production systems: From theory to practice. Science of The Total Environment, 735: 139378. Available from http://www.sciencedirect.com/science/article/pii/S0048969720328953. DOI https://doi.org/10.1016/j.scitotenv.2020.139378. 10.1016/j.scitotenv.2020.139378 PMC7374405 32480148

[b0415] Zuffo, A.M., F. Steiner, J.G. Aguilera, P.E. Teodoro, L.P.R. Teodoro and A. Busch, 2020. Multi-trait stability index: A tool for simultaneous selection of soya bean genotypes in drought and saline stress. Journal of Agronomy and Crop Science, 206(6): 815-822. Available from https://onlinelibrary.wiley.com/doi/abs/10.1111/jac.12409. DOI https://doi.org/10.1111/jac.12409.

